# 

**Published:** 1845-11

**Authors:** 


### CONTENTS

OF THE

### MEDICAL EXAMINER.

NEW SERIES—NO. XI.—NOVEMBER, 1845.

### ORIGINAL COMMUNICATIONS.

Case of Twins, in which one child was black, and the other white. By Alexander Cunningham, M. D. Communicated in a letter to Professor Dunglison, - - - - - - - - - - - 647

Case of Congenital Cataract in both eyes, one operated on successfully. By George Dock, M.D., of Harrisburg, Pennsylvania. Communicated in a letter to the editor, - - - - - - - 651

### BIBLIOGRAPHICAL NOTICES.

Elements of Materia Medica and Therapeutics. By John P. Harrison, M. D., Professor of Materia Medica and Therapeutics in the Medical College of Ohio, - - - - - - - - - - 652

A Dictionary of Terms used in Medicine and the Collateral Sciences. By Richard D. Hoblyn. A. M., Oxon. First American from the second London edition. Revised with numerous additions, by Isaac Hays, M. D., Editor of the American Journal of the Medical Sciences, - - - - - - - - - - - 659

The Domestic management of the Sick-Room, necessary, in aid of medical treatment, for the cure of Diseases. By Anthony Todd Thompson, M. D., F. L. S., etc. etc. First American from the second London edition. Revised, with additions, by R. E. Griffith, M.D. &c., &c. - - - - - - - - - - - - 662

A Practical Treatise on the Diseases of Children. By James Stewart, M. D., A. M., Fellow of the College of Physicians and Surgeons of New York, &c. &c. Third edition, carefully revised and enlarged, - - - - - - - - - - - - 663

EDITORIAL.

Health of Philadelphia, - - - - - - - - - - 664

Explosion of Saltpetre, - - - - - - - - - - 664

Medical Classes, - - - - - - - - - - - - 665

RECORD OF MEDICAL SCIENCE.

Report on the Progress of Practical Medicine, Pathology, and Therapeutics. By W. H. Ranking, M. D.

§ I.—Zymotic Diseases, - - - - - - - - - - 666

§ II.—Diseases of the Nervous System, - - - - - - 669

§ III.—Diseases of the Respiratory System, - - - - 675

§ IV.—Diseases of the Circulating System, - - - - - - 683

§ V.—Chylopoietic System, - - - - - - - - - 685

§ VI.—Genito-urinary System, - - - - - - - - - 687

§ VII.—Diseases of Uncertain seat, - - - - - - - 690

Sulphate of Quinine not absorbed when applied Endermically. By M. Martin-Solon, - - - - - - - - - - - 695

Case of Monstrosity. By M. Nicolas, - - - - - - - - 695

Sudden death from spontaneous rupture of the stomach. By Sig. Morici, - - - - - - - - - - - 695

Recovery from Wound of the Head, with loss of substance of the Brain. By M. Rouelle, - - - - - - - - - - 696

On the Causes of Plague. By M. Hamont, - - - - - 696

Escape of a Tænia solium through the Umbilicus. By M. Siebold, - 696

Experiments on the Mass of Blood relative to the Weight of the Body. By M. Wanner, - - - - - - - - - - 697

Experiments on the Blood. By M. Dupuy, - - - - - 698

Case of ulcerated Stomach, causing death by being suddenly detached from its adhesions to the peritoneal lining of the abdomen. By William Collins, surgeon, - - . - - - - 699

Case of Placenta Prævia. By J. C. Parker, Surgeon to the Bridgewater Infirmary, &c. - - - - - - - - - 700

Subcutaneous Punctures, - - - - - - - - - - 700

Embalmment by Injection, - - - - - - - - - 701

Extra-Uterine Pregnancy—Removal of the child by the Cæsarean operation. By M. M'Culloch, M. D. - - - - - - - - 701

On the Deposition of Carbonaceous Matter in the Tissue of the Lungs, 703

Transactions of the College of Physicians of Philadelphia, - - - 704

On the dressing of wounds by occlusion, - - - - - - - - 710

